# Clinical significance of interleukin-6, total bilirubin, CD3 + CD4 + T cells counts in the acute exacerbation of connective tissue disease-associated interstitial lung disease: a cross-sectional study

**DOI:** 10.1186/s40001-023-01384-0

**Published:** 2023-09-29

**Authors:** Chengxing Ma, Kaifang Meng, Shenyun Shi, Tingting Zhao, Shanshan Chen, Xuan Zhou, Ruilu Shu, Miao Ma, Mi Tian, Jingjing Ding

**Affiliations:** 1 Department of Respiratory and Critical Care Medicine, Nanjing Drum Tower Hospital, The Affiliated Hospital of Nanjing University Medical School, No. 321 Zhongshan Road, Nanjing, 210008 China; 2 Department of Respiratory and Critical Care Medicine, Nanjing Drum Tower Hospital Clinical College of Nanjing Medical University, Nanjing, China; 3 Department of Rheumatology, Nanjing Drum Tower Hospital, The Affiliated Hospital of Nanjing University Medical School, Nanjing, China; 4 Phase I Clinical Trials Unit, Nanjing Drum Tower Hospital, The Affiliated Hospital of Nanjing University Medical School, Nanjing, China

**Keywords:** Connective tissue disease-associated interstitial lung disease (CTD-ILD), Cytokines, Acute exacerbation (AE), Interleukin-6 (IL-6)

## Abstract

**Objective:**

Interstitial lung disease (ILD) is a severe complication of connective tissue disease (CTD) that can significantly impact patients' prognosis and quality of life. However, the current diagnostic arena lacks reliable biomarkers for detecting and monitoring the progression and exacerbation of CTD-ILD. This study aimed to investigate the clinical value of 12 serum cytokines in the diagnosis of CTD-ILD and prediction of the risk of acute exacerbation (AE) in this disease.

**Methods:**

This study was a cross-sectional investigation. Ninety-one hospitalized CTD patients were allocated into two groups: CTD-ILD group (*n* = 61) and CTD-non-ILD group (*n* = 30), and 30 sex-age matched healthy volunteers were enrolled as controls. The serum concentrations of interferon (IFN)-α, IFN-γ, tumor necrosis factor (TNF)-α, interleukin (IL)-2, IL-4, IL-5, IL-6, IL-8, IL-10, IL-12p70, IL-17A, and IL-1β were measured by Luminex suspension arrays. Logistic regression was employed to determine the significance of variables in the occurrence of AE-CTD-ILD. A nomogram was constructed to visualize the independent variables.

**Results:**

Elevated levels of IL-6, IL-8, and TNF-α were observed and compared in the CTD-ILD group with CTD-non-ILD (all *P* < 0.05). Similarly, the levels of IL-6, IL-8 and TNF-α were higher in the acute exacerbation (AE-CTD-ILD) group compared with stable CTD-ILD (S-CTD-ILD) (*P* < 0.001, *P* < 0.001, and *P* = 0.022). Significant correlations between serum IL-6 and PaO2/FiO2 ratio (*r* = − 0.463, *P* < 0.001), percent predicted forced vital capacity (FVC%; *r* = − 0.362, *P* < 0.05), and total ground-glass opacity (GGO) score (*r* = 0.439, *P* < 0.001) were observed in CTD-ILD patients. Multivariate logistic regression analysis revealed that elevated IL-6 levels, total bilirubin (TBil), and decreased CD3 + CD4 + T cells counts were independent risk factors for the occurrence of AE-CTD-ILD (OR = 1.121, *P* = 0.024; OR = 1.865, *P* = 0.047; OR = 0.983, *P* = 0.037, respectively). Furthermore, by employing these three variables in combination for the prediction of AE status, their collective impact surpasses the independent effects of any single biomarker.

**Conclusions:**

Elevated levels of serum IL-6, IL-8, and TNF-α were associated with the complication of ILD in CTD patients and the occurrence of AE in CTD-ILD patients. IL-6 could be a promising serum biomarker of severity and the occurrence of AE in CTD-ILD patients. The combination of the three variables (IL-6 level, TBil and CD3 + CD4 + T cells) predicted the AE-CTD-ILD better.

## Introduction

Interstitial lung disease (ILD) is a common and clinically important complication in most types of connective tissue disease (CTD), such as primary Sjögren's syndrome (pSS), polymyositis or dermatomyositis (PM/DM), undifferentiated connective tissue disease (UCTD), and rheumatoid arthritis (RA) [1]. The median survival time for patients with CTD-ILD is approximately 6.5 years, and the mortality rate of CTD caused by ILD is around 123.6/1000 person-years [2, 3]. However, the progression and prognosis of CTD-ILD vary greatly among individuals. Acute exacerbation (AE) was initially reported in patients with idiopathic pulmonary fibrosis (IPF), characterized by the development of pathological diffuse alveolar damage (DAD) upon ILD, which was clinically presented as sudden aggravation of dyspnea and new bilateral ground-glass opacity (GGO) or consolidation on chest imaging [4, 5]. The annual incidence of AE-IPF is as high as 20%, with median survival after AE-IPF generally no more than 3 months. The in-hospital mortality after AE-IPF usually exceeds 50% [6, 7]. Recently, AE was also confirmed to occur in CTD-ILD, which shared a similar incidence with IPF [8]. Moreover, CTD-ILD was most frequent in RA, and the in-hospital mortality of AE-CTD-ILD was as high as in patients with AE-IPF [9–11]. Therefore, reliable serum biomarkers in CTD-ILD for a more accurate evaluation of the risk of AE are a dire need to combat the disease.

Previous studies described that both pro-inflammatory (IL-6, IL-8, TGF-α) and anti-inflammatory (IL-10) cytokines were closely related to the complication of ILD in pSS and PM/DM [12, 13]. Moreover, serum interferon (IFN)-β, IL-6, and IL-10 were highly correlated with the occurrence of acute/subacute interstitial pneumonia (A/SIP) in PM/DM [14], and elevated IL-8 levels along with lower ratios of IL-4 to IFN-γ may contribute to the occurrence of anti-melanoma differentiation-associated gene 5 antibody (anti-MDA5)-positive PM/DM-ILD [13]. The mechanisms and etiology of AE are poorly understood. However, much evidence highlighted the importance of DAD and aggravated epithelial injury during the progression of AE [15, 16]. Analysis of inflammatory cells in bronchoalveolar lavage fluid (BALF) in patients with CTD-ILD revealed aggregation of neutrophils with or without increased percentages of lymphocytes, with neutrophils considered important effector cells that are associated with poor prognosis of CTD-ILD [17, 18]. Immunohistochemical analysis showed the increased CD4 + T lymphocytes in lung tissue were characteristic of RA-ILD [19]. The immune cell density in the bronchiolar interstitium from ILD was increased significantly, such as macrophages, CD4 + T lymphocytes, and neutrophils [20], which may synthesize these cytokines in patients with severe ILD.

Therefore, we investigated the panel of 12 cytokines profiles in CTD-ILD patients with or without ILD and further evaluated their potential roles as indicators of disease severity and the occurrence of AE in CTD-ILD patients. We present the following article in accordance with the STARD reporting checklist.

## Material and methods

### Study subjects

A total of 61 CTD-ILD and 30 CTD-non-ILD patients hospitalized in Nanjing Drum Tower Hospital between February 2020 and November 2021 were retrospectively included in the study. In the Center of Physical Examination, 30 healthy subjects with no evidence of comorbidities were included as controls. The ACR/EULAR criteria were used to diagnose pSS and RA [21, 22]. The diagnosis of DM/PM was according to Peter and Bohan's criteria [23]. UCTD was diagnosed if there was evidence of polyarthritis not satisfying ACR criteria or manifestations that did not fulfill criteria for specific rheumatic diseases [24]. High-resolution computed tomography (HRCT) images determined the complication with ILD. The definition of AE was according to the updated criteria of AE-IPF [5], modified slightly for application to CTD-ILD [8]. Briefly, (1) concurrent or previous diagnosis of CTD-ILD; (2) worsening of dyspnea typically in the past month; (3) HRCT showing new bilateral GGO and/or consolidation superimposed on the typical background of ILD; (4) deterioration cannot be explained entirely through fluid overload or heart failure. Medical records were reviewed to exclude participants with (1) a history of malignancy or severe lung diseases such as COPD, tuberculosis, and pulmonary embolism; (2) Other ILD, such as idiopathic interstitial pneumonia, drug toxicity or occupational or environmental exposure associated with ILD. All participants provided informed consent and agreed to utilize their serum for research. The ethics committee of Nanjing Drum Tower Hospital approved the study, and was conducted according to the principles of the Declaration of Helsinki (as revised in 2013).

### Data collection

Clinical data were collected retrospectively from medical records. Pulmonary function tests were performed based on ATS guidelines, and the values were expressed as percentages of the normal predicted values. The peripheral blood level counts of CD3 + CD4 + T cells and CD3 + CD8 + T cells were evaluated by flow cytometry (Beckman Coulter, USA), and the data were analyzed using Cytomics FC500 (Beckman Coulter, USA).

### Measurement of cytokines

Blood was collected using conventional methods and centrifuged immediately. The serum was aliquoted and frozen at − 80 °C until analysis. According to the manufacturer's instructions (Merck Millipore, Germany), Luminex suspension arrays were used to quantitatively detect serum biomarkers [IL-2, IL-4, IL-5, IL-6, IL-8, IL-10, IL-12P70, IL-17A, TNF-α, IFN-α, IFN-γ, and IL-1β]. All assays were performed in duplicate, and the median values were reported.

### Evaluation of HRCT scoring

The severity of ILD evaluated by HRCT scans was assessed independently by two well-trained pulmonary radiologists according to previous guidelines [25]. The extent of alveolar abnormalities was quantified by GGO score: 0: no GGO; 1: GGO ≤ 5% (abnormal but minimal); 2: GGO 6%–24%; 3: GGO 25%–49%; 4: GGO 50%–75%; 5: GGO > 75%. The extent of interstitial abnormality was quantified by fibrosis score: 0, no fibrosis; 1: no honeycombing but interlobular septal thickening; 2: honeycombing involving < 25%; 3: honeycombing 25–49%; 4: honeycombing 50–75%; 5: honeycombing > 75%. The average score of each lobe was summed as the total HRCT score.

### Statistical analysis

**Fig. 1:**
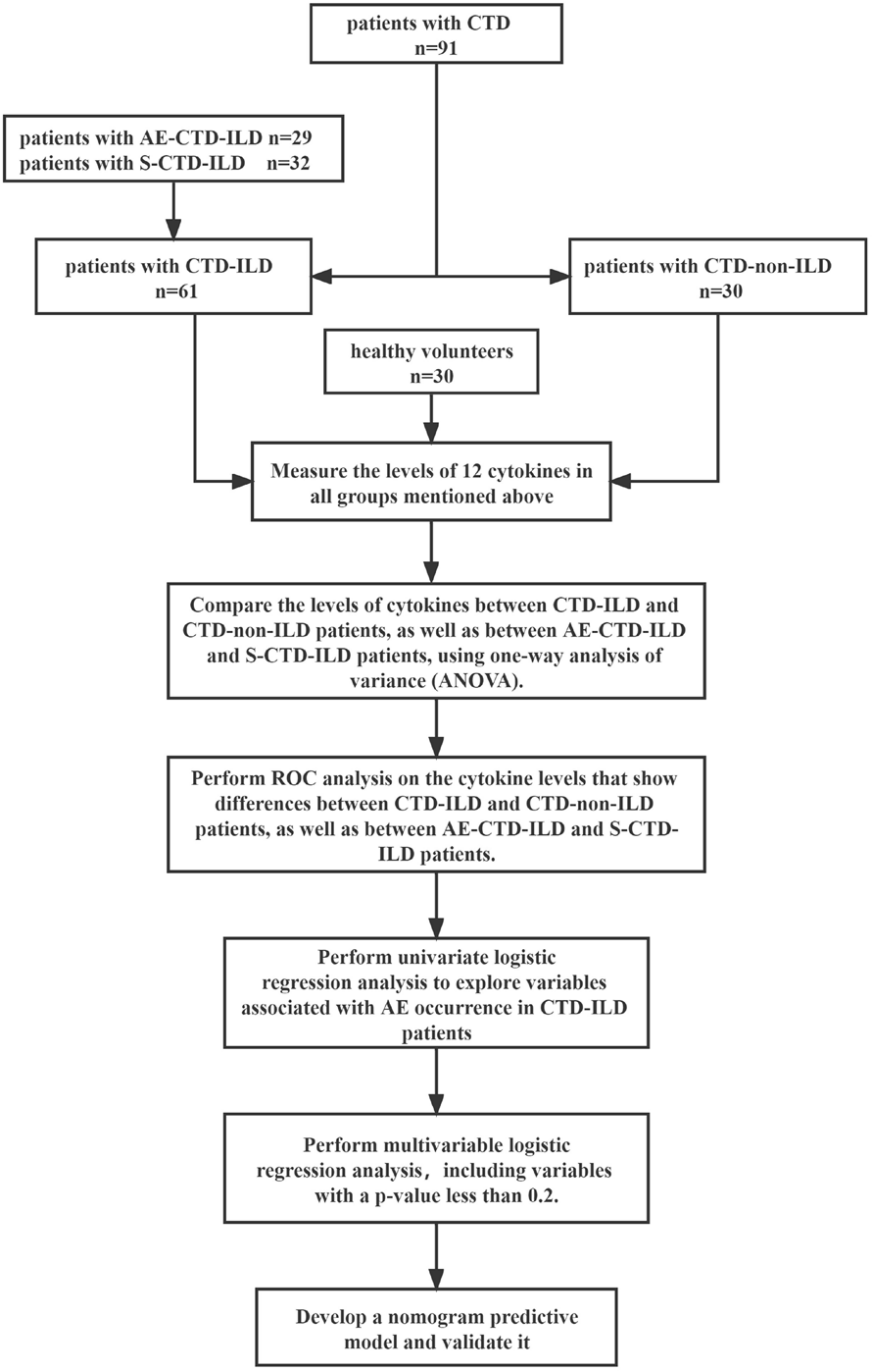
The flow diagram of the study

Statistical analysis for the frequency comparison was performed using the Chi-square test or Fisher's exact test, and the Mann–Whitney *U* test was used to compare continuous variables. The Spearman rank test evaluated correlations. Receiver operator characteristic (ROC) analyses were performed to calculate the area under the ROC curves (AUCs) of cytokines for discriminating CTD-ILD from CTD-non-ILD and AE-CTD-ILD from S-CTD-ILD. Logistic regression was employed to determine the significance of variables in the occurrence of AE-CTD-ILD. Based on R 4.2.1, a nomogram was constructed to visualize the independent variables selected through logistic regression analysis. The model's discrimination, calibration, and clinical utility were evaluated using the area under the ROC curve (AUC), calibration curve, and decision curve analysis (DCA) curve, respectively. GraphPad Prism version 8 (GraphPad Software Inc., La Jolla, CA, USA) was used for statistical analysis and plotting. *P* < 0.05 was defined as statistically significant. The flow diagram of this study is shown in Fig. 1.

## Results

### Clinical characteristics of the enrolled patients

The baseline clinical features of 61 CTD-ILD patients, including pSS (*n* = 20), PM/DM (*n* = 14), UCTD (*n* = 15), RA (*n* = 12), 30 CTD-non-ILD patients including pSS (*n* = 6), PM/DM (*n* = 7), UCTD (*n* = 13), RA (*n* = 4), and 30 healthy controls are summarized in Table 1. The median age in the CTD-ILD group was significantly higher than in CTD-non-ILD patients (*P* < 0.001). The serum level of total bilirubin (TBil) and systolic pulmonary artery hypertension (sPAH) were both significantly higher in the CTD-ILD group compared to the CTD-non-ILD group (*P* < 0.05). Severely decreased values of the percent predicted diffusing capacity of the lung for carbon monoxide (DLCO%) and percent predicted forced vital capacity (FVC%) were generally observed in CTD-ILD. In addition, HRCT scores revealed the alveolar abnormalities to be more prominent than interstitial abnormalities, with a relatively high median GGO score, possibly because the large number of CTD-ILD patients with AE were included in our study.

**Table 1:** Baseline clinical characteristics of patients with CTD-ILD, CTD-non-ILD and HCs

Clinical characteristics	HCs (*n* = 30)	CTD-non-ILD (*n* = 30)	CTD-ILD (*n* = 61)	p value (CTD-ILD vs. CTD-non-ILD)
Age, years	57.5 (46–63)	42.5 (28.8–63.0)	61.0 (53–68.5)	< 0.001***
Male, *n* (%)	8 (26.7)	6 (20)	17 (28)	0.417
Smoking history, *n* (%)	3 (10)	2 (15)	10 (16)	0.324
Prior immunosuppressant use, *n* (%)	N/A	21 (70)	39 (63.9)	0.566
LDH, U/L	N/A	245.0 (183.8–310.0)	285.0 (216.5–339.0)	0.111
CRP, mg/L	N/A	7.2 (2.6–21.3)	6.5 (3.6–22.0)	0.976
ESR, mm/h	N/A	37.0 (15.8–67.0)	37.0 (15.0–56.0)	0.780
WBC count, × 10^9^/L	N/A	5.9 (3.8–8.8)	6.7 (4.8–9.8)	0.253
Lymphocytes count, × 10^9^/L	N/A	1.1 (0.8–1.7)	1.2 (0.7–1.7)	0.879
NLR	N/A	3.8 (1.9–5.7)	4.4 (2.4–7.3)	0.265
RDW %	N/A	13.7 (12.60–14.5)	13.5 (12.7–14.4)	0.966
TBil, umol/L	N/A	5.7 (4.3–8.3)	7.1 (6.0–10.2)	0.046*
CD3 + CD4 + T cells, n/mm^3^	N/A	430.0 (218.0–535.0)	438.0 (245.0–725.0)	0.445
CD3 + CD8 + T cells, n/mm^3^	N/A	451 (285–1012)	432.0 (207.0–762.0)	0.244
sPAH, mmHg	N/A	27 (25–30)	32 (25–36)	0.001**
*Subtype of CTD*				
pSS, *n* (%)	N/A	6 (20.0)	20 (32.8)	0.204
PM/DM, *n* (%)	N/A	7 (23.3)	14 (23.0)	0.968
UCTD, *n* (%)	N/A	13 (43.3)	15 (24.6)	0.069
RA, *n* (%)	N/A	4 (13.3)	12 (19.7)	0.455
FVC %predicted	N/A	N/A	65.6 (42.9–83.6) (*n* = 30)	
DLCO %predicted	N/A	N/A	51.8 (42.1–73.2) (*n* = 30)	N/A
PaO2/FiO2 ratio	N/A	N/A	350 (286–414.3)	N/A
Total GGO score	N/A	N/A	19.0 (14.0–22.2)	N/A
Total fibrosis score	N/A	N/A	6.0 (5.0–10.7)	N/A

LDH, lactate dehydrogenase; NLR, neutrophil-to-lymphocyte ratio; RDW, red cell distribution width; sPAH, systolic pulmonary artery pressure; pSS, primary Sjögren's syndrome; PM/DM, polymyositis or dermatomyositis; UCTD, undifferentiated connective tissue disease; RA, rheumatoid arthritis; FVC: forced vital capacity; DLCO: diffusing capacity of the lung for carbon monoxide; PaO2/FiO2: partial arterial oxygen pressure/inspired oxygen fraction; GGO, ground-glass opacity

*P < 0.05, **P < 0.01, ***P < 0.001

### ROC analysis for discriminating CTD-ILD from CTD-non-ILD based on the levels of cytokines

**Fig. 2:**
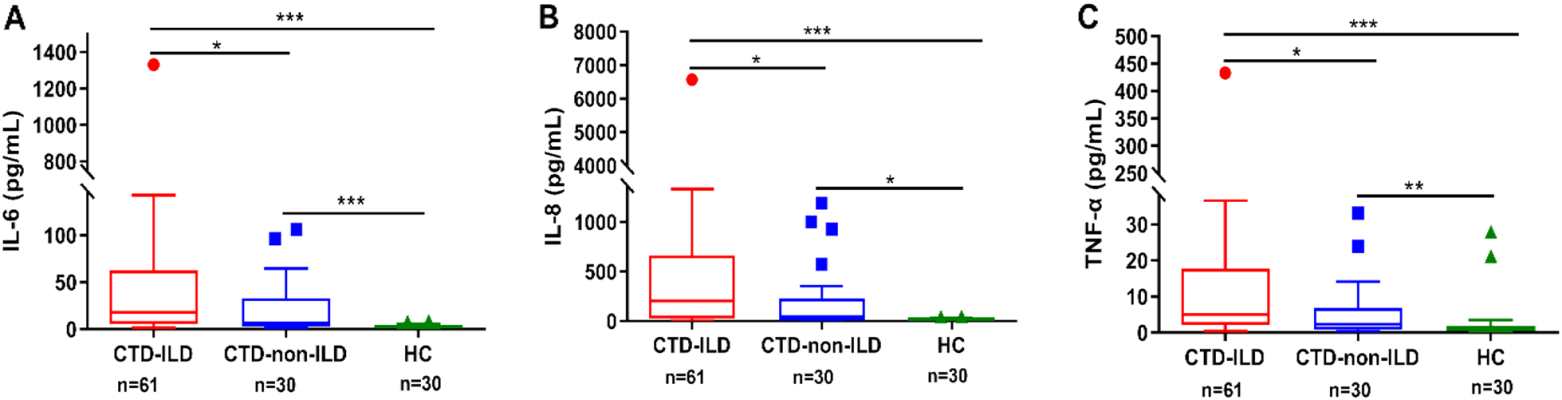
The levels of IL-6, IL-8, and TNF-α in CTD-ILD, CTD-non-ILD, and HCs. **A–C** Serum IL-6, IL-8, and TNF-α levels were significantly higher in patients with CTD-ILD than in CTD-non-ILD (all *P* < 0.05). *P < 0.05, **P < 0.01, ***P < 0.001

**Fig. 3:**
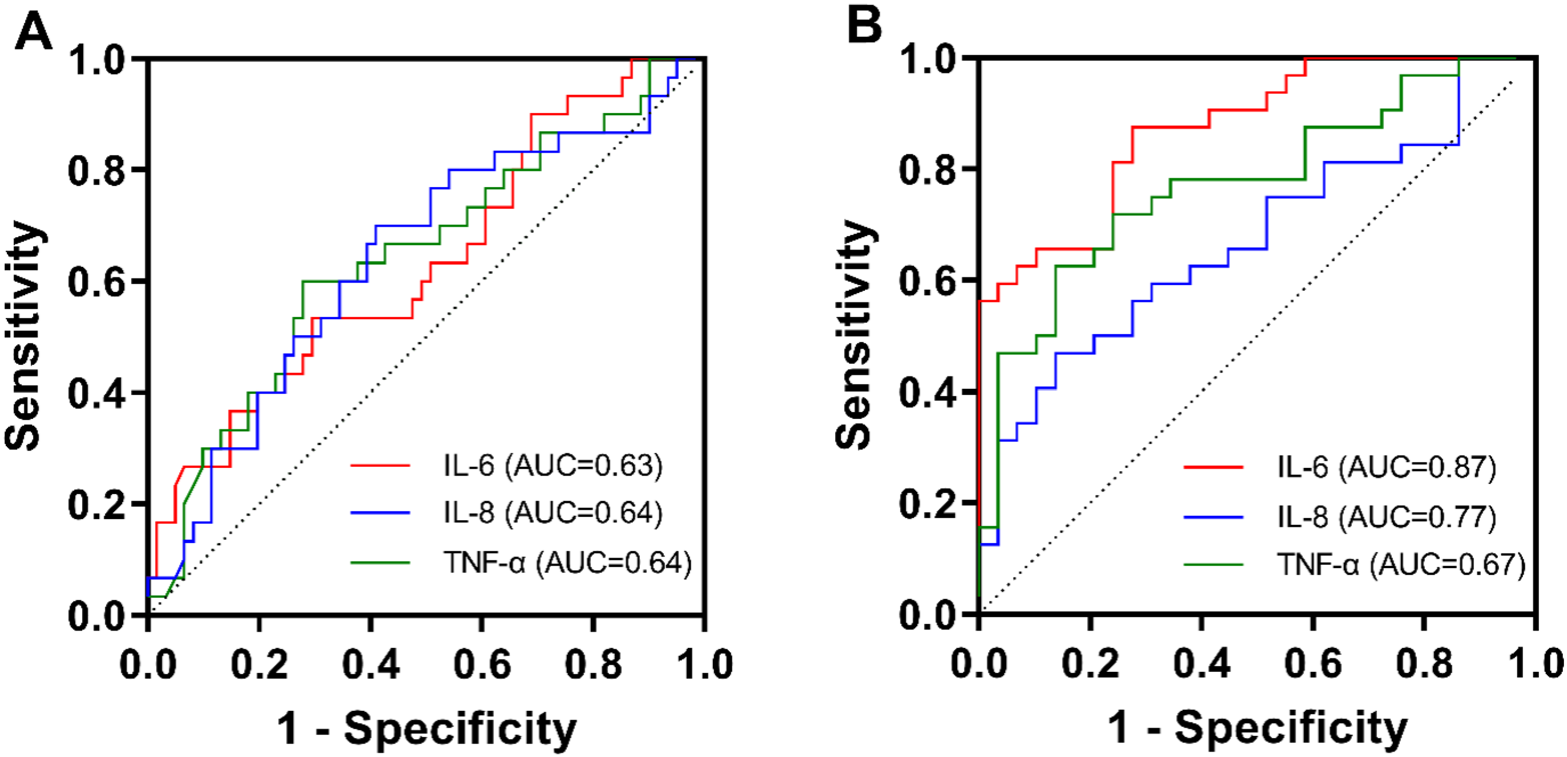
The areas under the ROC curves of IL-6, IL-8, and TNF-α. **A** The AUCs for differentiating CTD-ILD from CTD-non-ILD according to levels of IL-6, IL-8, and TNF-α were 0.63 (95% CI 0.51–0.75, *P* = 0.04), 0.64 (95% CI 0.52–0.76, *P* = 0.03) and 0.64 (95% CI 0.52–0.77, *P* = 0.02). **B** The AUCs for differentiating AE-CTD-ILD from S-CTD-ILD according to levels of IL-6, IL-8, and TNF-α were 0.87 (95% CI 0.79–0.96, *P* < 0.001), 0.77 (95% CI 0.66–0.89, *P* < 0.001), and 0.67 (95% CI 0.54–0.81, *P* = 0.020)

The cytokine profiles of all patients with CTDs were determined by evaluating the levels of each cytokine in CTD-ILD, CTD-non-ILD, and healthy controls (Table 2). The levels of 12 cytokines were significantly higher in both CTD-non-ILD and CTD-ILD groups than in healthy controls (all *P* < 0.05). Elevated levels of IL-6 (*P* = 0.042), IL-8 (*P* = 0.031), and TNF-α (*P* = 0.026) were also observed in CTD-ILD compared with CTD-non-ILD (Fig. 2). The accuracy of IL-6, IL-8 and TNF-α for differentiating CTD-ILD from CTD-non-ILD was further evaluated by ROC analyses, and the AUCs were 0.63 (95% CI 0.51–0.75, *P* = 0.04), 0.64 (95% CI 0.52–0.76, *P* = 0.03) and 0.64 (95% CI 0.52–0.77, *P* = 0.02; Fig. 3a).

**Table 2:** Comparison of cytokine levels among CTD-ILD, CTD-non-ILD and HCs

Variables	HCs (*n* = 30), pg/mL	CTD-non-ILD (*n* = 30), pg/mL	CTD-ILD (*n* = 61), pg/mL	p value (CTD-ILD vs. CTD-non-ILD)
IL-2	0.4 (0.3–0.8)	0.9 (0.4–1.6)*	1.0 (0.6–1.7)^###^	0.371
IL-4	0.5 (0.1–0.9)	1.4 (1.0–2.5)***	1.5 (0.9–2.8)^###^	0.645
IL-5	0.1 (0–0.5)	0.7 (0.3–1.0)***	0.8 (0.3–1.3)^###^	0.285
IL-6	2.2 (1.3–3.7)	6.7 (2.4–32.9)***	17.8 (5.4–62.5)^###^	0.042
IL-8	12.5 (8.3–20.9)	44.6 (6.7–226.2)*	203.2 (25.8–663.5)^###^	0.031
IL-10	1.2 (0.8–1.5)	4.1 (2.9–6.0)***	4.7 (3.1–6.6)^###^	0.510
IL-1β	0.3 (0–1.0)	4.3 (1.6–11.8)***	6.6 (2.3–12.6)^###^	0.329
IL-17A	2.1 (1.1–3.7)	11.9 (4.3–19.0)***	9.5 (5.3–16.6)^###^	0.543
IL-12p70	1.5 (0.7–2.1)	2.4 (1.3–3.1)**	2.8 (1.8–4.7)^###^	0.113
TNF-α	0.9 (0.4–1.9)	2.3 (0.9–6.8)**	5.0 (2.2–17.7)^###^	0.026
IFN-α	0.4 (0–1.0)	2.4 (1.5–7.0)***	2.4 (1.6–4.4)^###^	0.923
IFN-γ	0.8 (0.5–1.2)	2.1 (1.5–3.9)***	2.4 (1.6–4.6)^###^	0.445

CTD-ILD, connective tissue disease-associated interstitial lung disease; HCs, healthy controls

*P < 0.05 (HCs vs. CTD-non-ILD), **P < 0.01 (HCs vs. CTD-non-ILD), ***P < 0.001 (HCs vs. CTD-non-ILD), ^###^*P* < 0.001 (HCs vs. CTD-ILD)

### ROC analysis for discriminating AE-CTD-ILD from S-CTD-ILD based on the levels of cytokines

**Fig. 4:**
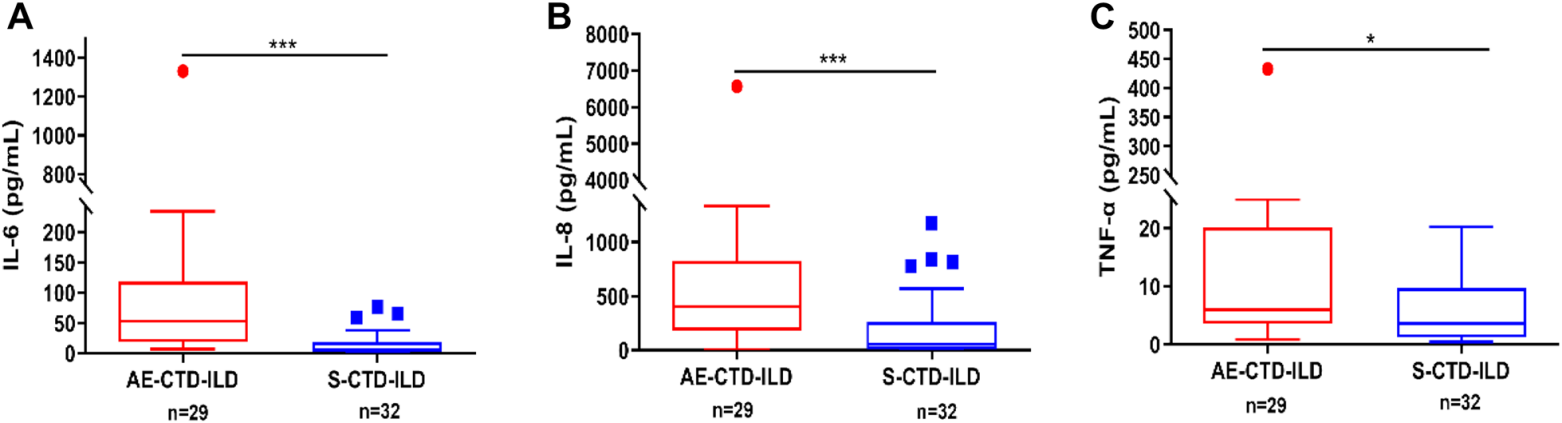
The levels of IL-6, IL-8, and TNF-α in AE-CTD-ILD and S-CTD-ILD. **A**–**C** Serum IL-6, IL-8, and TNF-α levels were significantly higher in the patients with AE-CTD-ILD than in S-CTD-ILD (*P* < 0.001, *P* < 0.001, and *P* = 0.022, respectively). *P < 0.05, **P < 0.01, ***P < 0.001

Among CTD-ILD patients, there were 29 with AE-CTD-ILD and 32 with S-CTD-ILD. To reveal the possible role of cytokine profiles in the pathogenesis of AE in CTD-ILD patients, cytokine levels were compared between the two subsets (Table 3). Figure 4 shows elevated levels of IL-6, IL-8, and TNF-α were observed in AE-CTD-ILD compared with the S-CTD-ILD (*P* < 0.001, *P* < 0.001, and *P* = 0.022, respectively). ROC was further analyzed to identify AE-CTD-ILD from S-CTD-ILD. The AUCs of IL-6, IL-8 and TNF-α were determined as 0.87 (95% CI 0.79–0.96, *P* < 0.001), 0.77 (95% CI 0.66–0.89, *P* < 0.001), and 0.67 (95% CI 0.54–0.81, *P* = 0.020), respectively (Fig. 3b).

**Table 3:** Comparison of cytokine levels between AE-CTD-ILD and S-CTD-ILD

Variables	S-CTD-ILD (*n* = 32), pg/mL	AE-CTD-ILD (*n* = 29), pg/mL	*P* value
IL-2	0.9 (0.6–1.3)	1.0 (0.7–2.4)	0.233
IL-4	1.4 (0.9–2.7)	1.8 (1.1–3.0)	0.583
IL-5	0.8 (0.3–1.2)	0.9 (0.4–1.5)	0.435
IL-6	5.6 (3.6–18.6)	53.2 (18.6–118.3)	< 0.001***
IL-8	56.9 (8.4–260.1)	402.9 (184.1–827.1)	< 0.001***
IL-10	4.5 (2.6–5.4)	5.7 (3.5–8.1)	0.070
IL-1β	5.2 (1.7–10.0)	8.6 (2.8–15.1)	0.147
IL-17A	9.4 (5.2–13.1)	13.0 (5.1–20.3)	0.220
IL-12p70	2.8 (1.4–5.1)	3.4 (1.8–4.5)	0.908
TNF-α	2.31 (0.88–6.82)	6.0 (3.6–20.1)	0.022*
IFN-α	2.1 (1.6–3.8)	2.9 (1.7–5.0)	0.201
IFN-γ	2.5 (1.5–5.0)	2.4 (2.0–4.6)	0.288

CTD-ILD, connective tissue disease-associated interstitial lung disease; AE, acute exacerbation

*P < 0.05, **P < 0.01, ***P < 0.001

### Correlations between clinical variables and cytokine levels in CTD-ILD patients

The correlations between IL-6, IL-8, and TNF-α levels and clinical variables of CTD-ILD are shown in Table 4. The levels of IL-6 were significantly correlated with total GGO score (*r* = 0.439, *P* < 0.001), ESR (*r* = 0.315, *P* < 0.05), FVC% (*r* = − 0.362, *P* < 0.05), and partial arterial oxygen pressure/inspired oxygen fraction (PaO2/FiO2) ratio (*r* = − 0.463, *P* < 0.001).

**Table 4:** Spearman correlations between clinical variables and cytokine levels in CTD-ILD patients

	LDH	ESR	CRP	PaO2/FiO2 ratio	FVC %	DLCO %	sPAH	Total GGO score	Total fibrosis score
IL-6	0.193	0.315*	0.235	− 0.463***	− 0.362*	− 0.164	0.07	0.439***	− 0.05
IL-8	0.064	0.04	− 0.02	− 0.115	− 0.036	0.213	0.062	0.239	0.013
TNF-α	− 0.018	0.136	0.199	− 0.154	− 0.309	− 0.041	− 0.069	0.085	0.017

LDH, lactate dehydrogenase; PaO2/FiO2: partial arterial oxygen pressure/inspired oxygen fraction; FVC: forced vital capacity; DLCO: diffusing capacity of the lung for carbon monoxide; sPAH, systolic pulmonary artery pressure; GGO, ground-glass opacity

### Predictive value of clinical characteristics and cytokines for AE in CTD-ILD patients

**Fig. 5:**
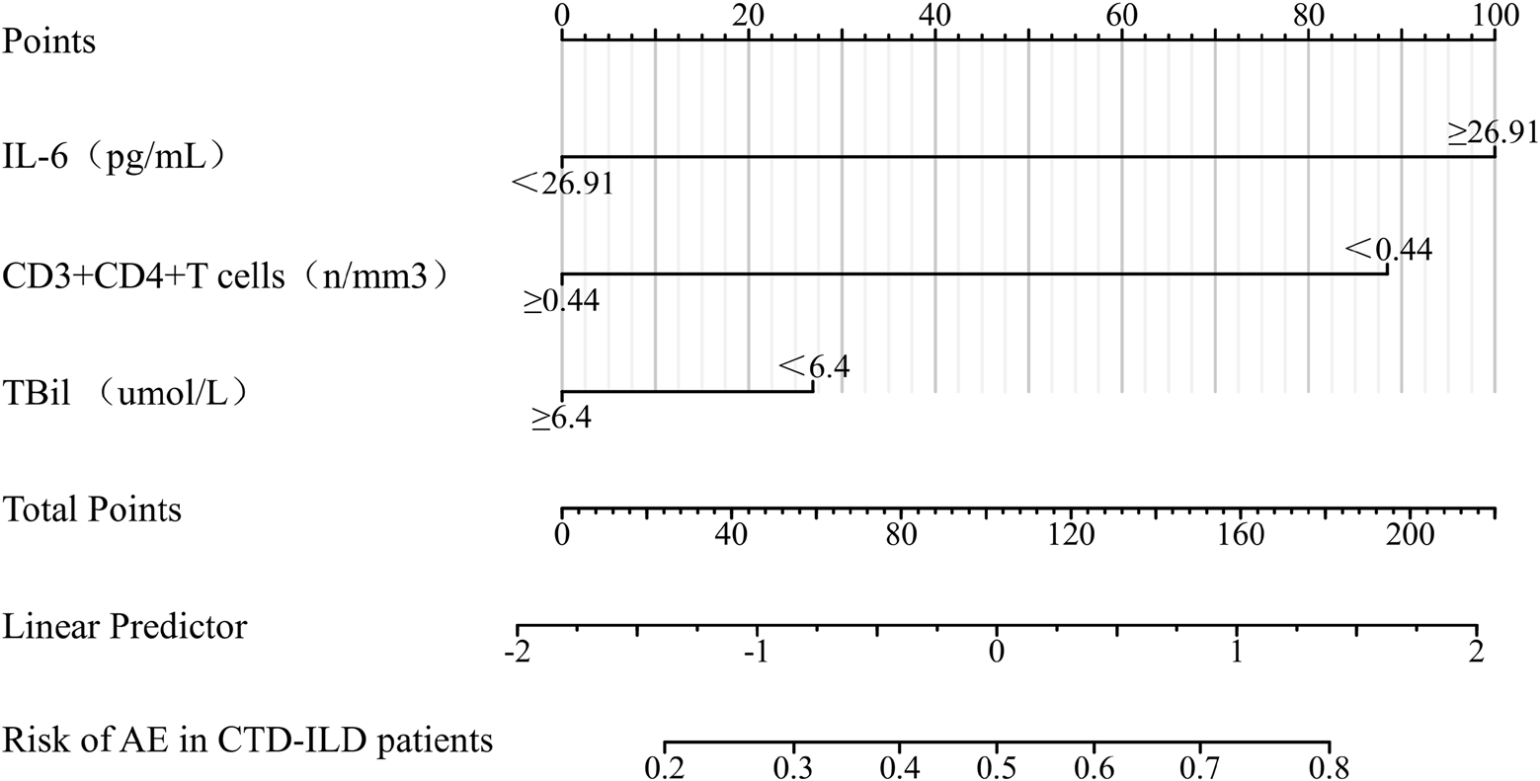
The nomogram prediction model for diagnosing the occurrence of AE in CTD-ILD patients

**Fig. 6:**
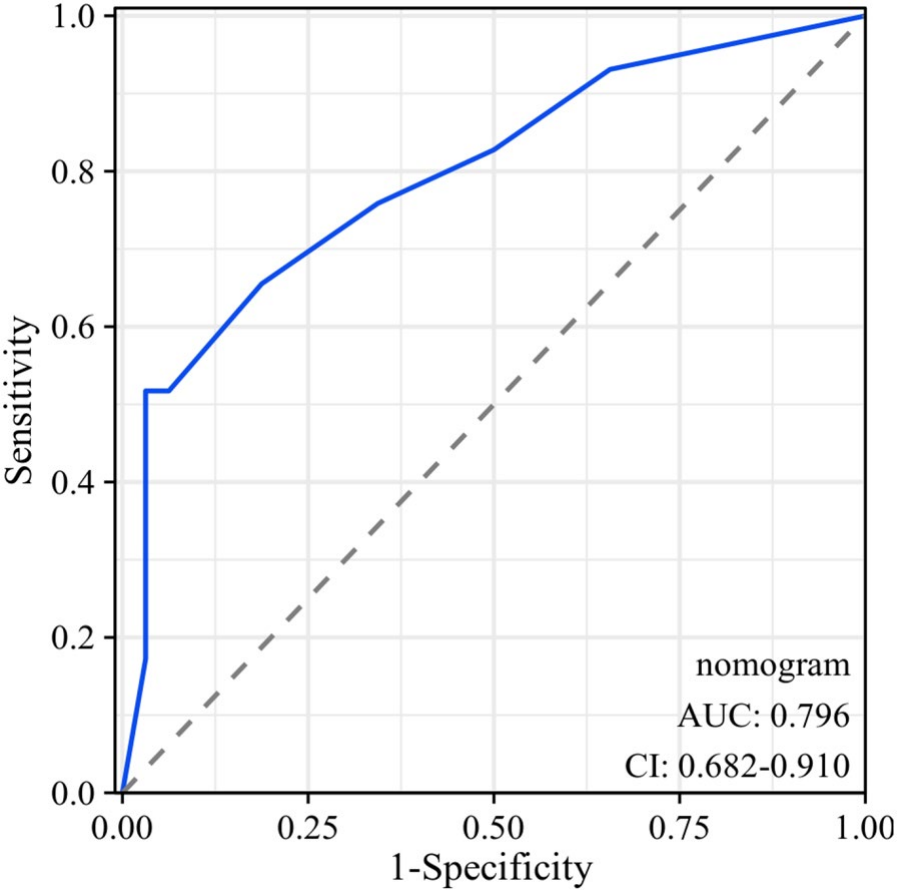
The areas under the ROC curves of the nomogram prediction model. The AUCs for differentiating AE-CTD-ILD from CTD-ILD according to this model were 0.796 (95% CI 0.682–0.910, *P* < 0.01)

**Fig. 7:**
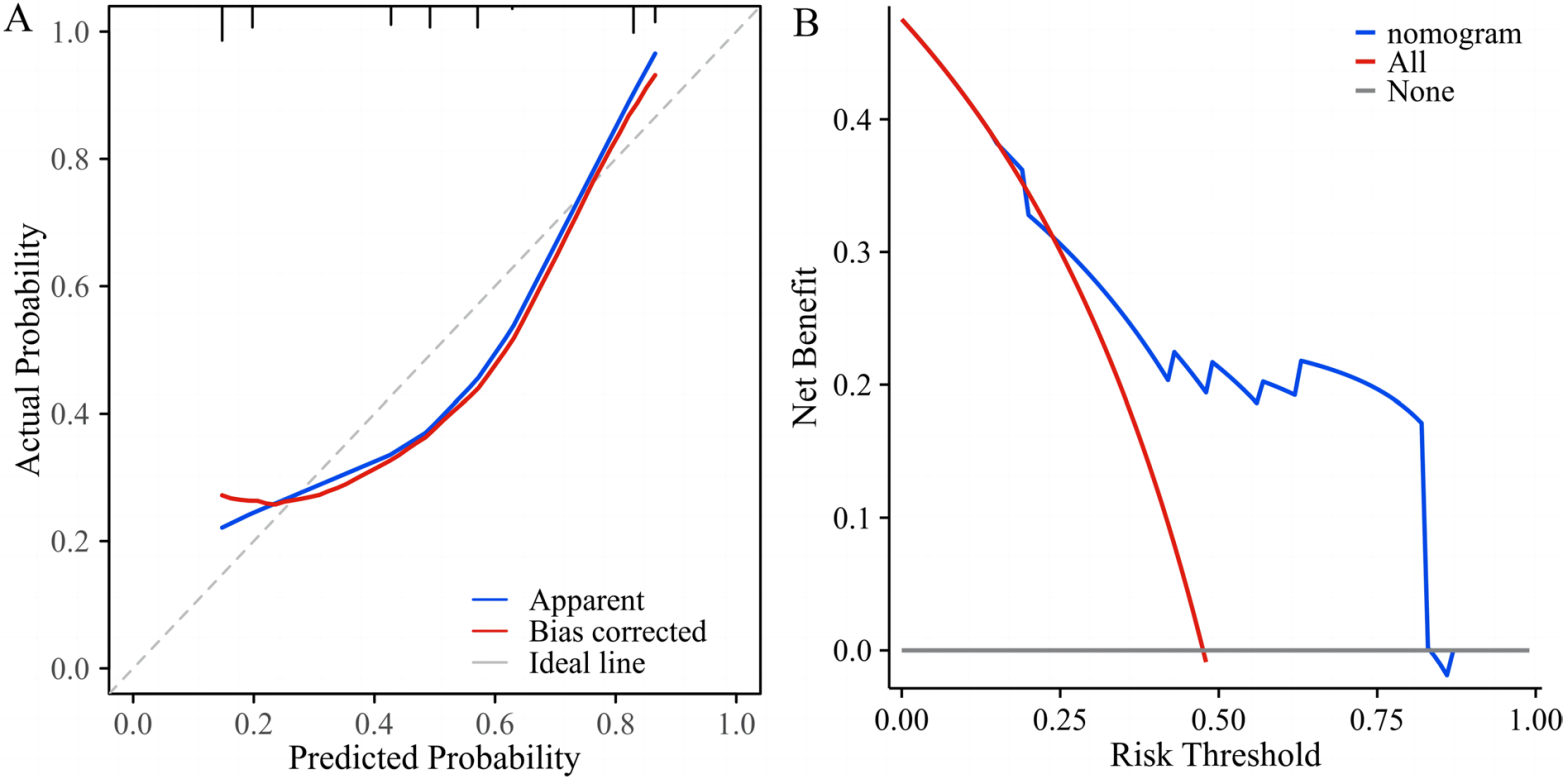
**a** The calibration curves of the AE-CTD-ILD diagnostic nomogram. The “Ideal line” represents the optimal predictive performance, while the “Apparent” represents the prediction probability curve fitted by the nomogram model. The “Bias correction” refers to the prediction probability curve after adjusting for overfitting. The “Apparent” represents the prediction probability curve fitted by the nomogram model, and the “Bias correction” represents the prediction probability curve after correcting for overfitting, thereby indicating the predictive performance of the nomogram model. **b** The decision curve of the nomogram model, serving as a tool to evaluate the practicality and benefits of the model. The decision curve analysis indicates that the developed nomogram in this study exhibits substantial benefits, suggesting a viable potential application value for clinical decision-making

Univariate and multivariate logistic regression analyses were performed to investigate the risk factors of AE occurrence in CTD-ILD patients. Univariate analysis demonstrated that the serum IL-6 (OR = 1.044, *P* < 0.001), IL-8 (OR = 1.002, *P* = 0.011), LDH (OR = 1.008, *P* = 0.007), along with the decreased values of CD3 + CD4 + T cells (OR = 0.998, *P* = 0.042) and PaO2/FiO2 ratio (OR = 0.986, *P* < 0.001) were associated with the occurrence of AE. Variables with p value less than 0.2 in univariate analysis were successfully included in multivariate regression analysis, in which elevated serum IL-6 level (OR = 1.121, *P* = 0.024), TBil (OR = 1.865, *P* = 0.047), and decreased values of CD3 + CD4 + T cells (OR = 0.983, *P* = 0.037) acted as the risk factors (Table 5). The nomogram prediction model was developed using the three variables combined above (Fig. 5). The combination of the three variables predicted AE condition better than any of the single indicators. The Omnibus test for this model yielded a significant result with *P* < 0.001, indicating its statistical significance. The Hosmer–Lemeshow test resulted in χ^2^ = 2.8975, *P* = 0.9406, suggesting a good fit of the predictive model. The area under the ROC curve for this predictive model in the modeling cohort was 0.796, (95% CI 0.682–0.910, *P* < 0.001; Fig. 6). Furthermore, in the diagnostic calibration curve of this predictive model, the HL goodness-of-fit test yielded a *P*-value of 0.952, indicating a strong fit of the model (Fig. 7a). The decision curve analysis revealed that the net benefit of using this model for predicting AE occurrences in CTD-ILD was higher compared to using any single cytokine marker alone (Fig. 7b).

**Table 5:** Variables associated with AE occurrence in CTD-ILD patients according to logistic regression analysis

Variables	Univariate analysis			Multivariate analysis		
	OR	95% CI	*P* value	OR	95% CI	*P* value
Age, years	1.011	0.967–1.057	0.640			
Male	1.350	0.440–4.146	0.600			
Smoking history	1.826	0.459–7.260	0.392			
IL-2	1.053	0.850–1.305	0.638			
IL-4	1.032	0.930–1.145	0.550			
IL-5	1.526	0.793–2.937	0.206			
IL-6	1.044	1.017–1.072	0.001***	1.121	1.015–1.239	0.024*
IL-8	1.002	1.001–1.004	0.011*	1.006	0.999–1.012	0.074
IL-10	1.076	0.946–1.224	0.264			
IL-1β	1.023	0.962–1.089	0.469			
IL-17A	1.027	0.985–1.070	0.218			
IL-12p70	0.997	0.832–1.195	0.976			
TNF-α	1.014	0.994–1.034	0.159	1.050	0.995–1.108	0.077
IFN-α	1.105	0.923–1.322	0.277			
IFN-γ	1.080	0.879–1.326	0.464			
LDH, U/L	1.008	1.002–1.014	0.007**	1.009	0.995–1.024	0.197
CRP, mg/L	1.013	0.995–1.031	0.161	0.959	0.914–1.006	0.086
ESR, mm/h	1.011	0.992–1.031	0.268			
NLR	1.102	0.975–1.245	0.120	0.809	0.354–1.850	0.616
RDW %	1.066	0.771–1.474	0.698			
TBil, umol/L	1.095	0.971–1.235	0.140	1.865	1.008–3.450	0.047*
CD3 + CD4 + T cells	0.998	0.996–1.000	0.042*	0.983	0.968–0.999	0.037*
sPAH, mmHg	1.017	0.965–1.072	0.527			
PaO2/FiO2 ratio	0.986	0.979–0.994	0.001***	0.985	0.966–1.004	0.123

LDH, lactate dehydrogenase; NLR, neutrophil-to-lymphocyte ratio; RDW, red cell distribution width; sPAH, systolic pulmonary artery pressure; PaO2/FiO2: partial arterial oxygen pressure/inspired oxygen fraction; GGO, ground-glass opacity. OR, odds ratio; CI, confidence interval

*P < 0.05, **P < 0.01, ***P < 0.001

### Comparison of cytokine levels between anti-MDA5-positive and anti-MDA5-negative subsets in PM/DM-ILD

In this study, the comparative analysis of the cytokines profile in 8 anti-MDA5-positive and 6 anti-MDA5-negative PM/DM-ILD patients was also performed (Table 6). The findings revealed that IL-4 and IL-12p70 levels in the subset of anti-MDA5-positive patients were significantly lower than in anti-MDA5-negative patients (*P* = 0.02 and *P* = 0.02). Intriguingly, IL-4 levels were significantly correlated with the levels of IL-12p70 (*r* = 0.613, *P* = 0.022).

**Table 6:** Comparison of cytokine levels between anti-MDA5-positive and anti-MDA5-negative patients in PM/DM

Variables	Anti-MDA5-positive PM/DM-ILD (*n* = 8), pg/mL	Anti-MDA5-negative PM/DM-ILD (*n* = 6), pg/mL	*P* value
IL-2	0.5 (0.2–1.3)	1.6 (1.1–2.4)	0.081
IL-4	1.1 (0.3–1.4)	3.2 (1.5–3.8)	0.020*
IL-5	0.4 (0.3–1.1)	1.1 (0.8–1.4)	0.181
IL-6	26.5 (8.8–58.1)	5.7 (5.2–12.3)	0.142
IL-8	197.2 (27.5–648.5)	41.8 (15.4–110.3)	0.181
IL-10	4.1 (3.2–6.8)	5.4 (4.5–8.1)	0.491
IL-1β	3.0 (2.2–6.7)	6.0 (4.1–7.9)	0.181
IL-17A	9.7 (6.6–13.1)	10.6 (6.1–22.8)	0.662
IL-12p70	2.3 (1.3–3.2)	5.0 (3.8–6.6)	0.020*
TNF-α	4.7 (1.5–18.2)	4.4 (3.0–5.8)	0.950
IFN-α	5.3 (1.5–10.4)	4.2 (2.0–8.5)	0.852
IFN-γ	3.1 (1.5–6.7)	5.4 (2.2–6.4)	0.414

MDA5, melanoma differentiation-associated gene 5; PM/DM-ILD, polymyositis/dermatomyositis associated interstitial lung disease

*P < 0.05

## Discussion

ILD is a clinically significant manifestation of CTD, with a more unfavorable course and prognosis [10]. The varying clinical nature makes AE fatal and challenging, raising morbidity and mortality rates. As such, identifying reliable biomarkers in CTD to monitor the progression of ILD and predict the onset of AE in CTD-ILD patients could be valuable in guiding treatment decisions. Several risk factors for AE in CTD-ILD patients have been reported, including advanced age [9], the presence of a UIP pattern on HRCT [10], and systemic sclerosis overlapping with PM/DM [26], but there still exists a knowledge gap in fully comprehending the underlying mechanism of the disease. Our study found that levels of IL-6, IL-8, and TNF-α were elevated in the CTD-ILD group compared to CTD-non-ILD and in the subset of AE-CTD-ILD compared to S-CTD-ILD. Additionally, elevated serum IL-6, total bilirubin, and decreased CD3 + CD4 + T cell counts were independent risk factors for AE in CTD-ILD patients.

The development of AE in patients with CTD-ILD is not a spontaneous event but rather is triggered by endogenous factors such as inflammation and disruptions in immune homeostasis. The pathogenesis of ILD is complex and involves multiple factors, including alveolar inflammation, alveolar epithelial cell injury, and cytokine release. Fluctuations in these factors can contribute to the progression of AE [27]. In IPF patients, AE is often characterized by a histopathological pattern known as DAD [15]. Several serum markers have been found to predict AE in IPF patients, including Krebs von den Lungen-6 (KL-6), sCD206, IFN-γ, and IL-6 [27–30]. Similar to IPF, DAD-pattern acute interstitial pneumonia can also occur in patients with PM/DM and RA [9]. However, the literature is sparsely populated with the pathogenesis of AE in CTD-ILD patients and validated predictors of AE-CTD-ILD are currently lacking.

IL-6 is a pro-inflammatory cytokine produced by macrophages, activated T cells, and fibroblasts in response to external stimuli during the acute phase of inflammation [31]. Notably, IL-6 can promote the differentiation of CD4 + cells into the pro-fibrotic Th2 type, activate the TGF-β pathway, suppress Th1 differentiation, and shift T cells from a regulatory response to a pathogenic Th17 response [32–34]. IL-6 gene knockout mice showed attenuated irradiation or bleomycin-induced pulmonary fibrosis [35]. Elevated levels of IL-6 were reported in the AE-IPF group compared to stable IPF in IPF patients [27, 30, 36, 37]. Our study showed that the serum concentrations of IL-6 were significantly elevated in patients with CTD-ILD compared to CTD-non-ILD. Higher IL-6 levels were associated with total GGO scores in CTD-ILD, suggesting that elevated IL-6 levels may reflect severe inflammation in CTD-ILD patients. ROC curve analysis revealed that higher serum IL-6 levels were useful in predicting ILD, consistent with a previous study in pSS [12]. Lee et al. [30] demonstrated that serum IL-6 might be utilized as a biomarker to predict AE in ILD with different diagnoses, including IPF (68.7%), CTD (14.5%), cryptogenic organizing pneumonia (9.6%), non-specific interstitial pneumonia (6.0%), and hypersensitivity pneumonitis (1.2%). Our study indicated that serum IL-6 was significant in identifying AE from stable ILD in CTD-ILD patients, in accordance with Lee et al. [30]. Although the role of IL-6 in CTD-ILD has not been elucidated, we speculated that the pro-inflammatory and pro-fibrotic characteristics of IL-6 are pivotal in the pathogenesis of CTD-ILD and its associated acute exacerbation (AE) state.

IL-8 is a critical inflammatory factor for the migration of chemotactic neutrophils to damaged tissue. Elevated levels of IL-8 in BALF and sputum were observed in IPF patients, which is indicative of future AE in IPF [38]. Serum IL-8 levels were also associated with the complication of ILD in pSS and PM/DM [12, 13], with higher IL-8 levels possibly contributing to the pathophysiology of anti-MDA5-positive ILD in PM/DM [13]. TNF-α is one of the cytokines produced by activated macrophages and mononuclear cells. It activates and recruits different inflammatory cells while coordinating the production of pro-inflammatory cytokine cascades in many inflammatory diseases [12]. In IPF patients, activated macrophages and TNF-α production play critical roles in the progression of pulmonary inflammation and/or fibrosis [39]. In our study, elevated levels of IL-8 and TNF-α were identified in CTD-ILD compared to CTD-non-ILD and AE-CTD-ILD compared to S-CTD-ILD. The possible explanation for this phenomenon is that pro-inflammatory cytokines may play crucial roles in the progression of interstitial lung injury and the pathogenesis of AE in CTD-ILD patients.

Previous research has suggested systemic T cell activation in organs may be related to the pathogenesis of RP-ILD in DM [40]. In the study by Gui et al., the decreased percentage of CD3 + CD4 + T cells was closely related to A/SIP onset in MDA5-DM patients [41]. Although the precise functions of CD3 + CD4 + T cells in CTD-ILD were not elucidated, we found that the decreased number of peripheral CD3 + CD4 + T cells was an independent risk factor for AE in CTD-ILD patients. A previous study has described the migration of lymphocytes to the lungs to participate in local immune responses, resulting in a peripheral decrease of lymphocytes [42]. We speculate that the reduced CD3 + CD4 + T cell count increases the risk of infection and thus contributes to the occurrence of AE. In addition, we observed elevated serum TBil in CTD-ILD compared with CTD-non-ILD, and higher serum TBil may be used as an independent risk factor for AE. However, further research is needed to better understand the role of serum TBil in the pathophysiology of AE-CTD-ILD. Based on three factors, IL-6, Tbil, and CD3 + CD4 + T cells, this study developed a nomogram model for AE-CTD-ILD. The predictive model demonstrates good fit and calibration, facilitating the effective assessment of the risk of acute exacerbation in CTD-ILD patients and enabling personalized preventive interventions.

Our study also found that IL-4 and IL-12p70 were significantly decreased in anti-MDA5-positive ILD compared to anti-MDA5-negative ILD in PM/DM. IL-4 is an anti-inflammatory cytokine mainly secreted by Th2 cells, and the ratio of serum IL-4 to IFN-γ usually reflects the balance between Th1 and Th2 cells. IL-12p70 is a pro-inflammatory cytokine in Th1 cell differentiation and IFN-γ production, essential for the induction of JAK–STAT signal transduction [43]. Our results suggesting a potential relationship between Th1/Th2 bias and the pathogenesis of anti-MDA5-positive DM are interesting, and further study on the relationship between IL-12 and JAK–STAT signal pathway in anti-MDA5-positive DM may provide valuable insights into the pathogenesis of this rare autoimmune disease.

Despite the strengths of our study design and methodology, it is important to acknowledge the limitation of our study. Firstly, this study was performed retrospectively on a small population from a single center, introducing selection bias. Secondly, patients with different subtypes of CTD were combined and classified into the same group, and the pathophysiology of different subtypes of CTD may be distinct, such as anti-MDA5-positive PM/DM. Finally, we did not perform a longitudinal analysis of cytokine profiles before and after treatment.

In light of these findings, we found that elevated levels of serum IL-6, IL-8, and TNF-α were associated with the development of ILD in CTD patients and the occurrence of AE in CTD-ILD patients. IL-4 and IL-12p70 levels in the anti-MDA5-positive subset were significantly lower than in the anti-MDA5-negative subset in PM/DM-ILD. Moreover, serum IL-6 levels, CD3 + CD4 + T cells, and TBil may serve as independent biomarkers for predicting the occurrence of AE in CTD-ILD. The combined predictive effect of these three variables surpasses that of any individual marker alone.

## Data Availability

The datasets used and/or analyzed during the current study are available from the corresponding author on reasonable request.
